# Large‐sized rare tree species contribute disproportionately to functional diversity in resource acquisition in African tropical forest

**DOI:** 10.1002/ece3.4836

**Published:** 2019-04-02

**Authors:** Elizabeth Kearsley, Koen Hufkens, Hans Verbeeck, Marijn Bauters, Hans Beeckman, Pascal Boeckx, Dries Huygens

**Affiliations:** ^1^ Department of Environment Ghent University Gent Belgium; ^2^ INRA UMR ISPA Villenave d'Ornon France; ^3^ Department of Green Chemistry and Technology Ghent University Gent Belgium; ^4^ Service of Wood Biology Royal Museum for Central Africa Tervuren Belgium; ^5^ Institute of Agricultural Engineering and Soil Science Universidad Austral de Chile Valdivia Chile

**Keywords:** aboveground carbon storage, abundance, functional redundancy, functional specialization, functional traits, rareness, tree size

## Abstract

Increasing evidence is available for a positive effect of biodiversity on ecosystem productivity and standing biomass, also in highly diverse systems as tropical forests. Biodiversity conservation could therefore be a critical aspect of climate mitigation policies. There is, however, limited understanding of the role of individual species for this relationship, which could aid in focusing conservation efforts and forest management planning. This study characterizes the functional specialization and redundancy for 95% of all tree species (basal area weighted percentage) in a diverse tropical forest in the central Congo Basin and relates this to species' abundance, contribution to aboveground carbon, and maximum size. Functional characterization is based on a set of traits related to resource acquisition (wood density, specific leaf area, leaf carbon, nitrogen and phosphorus content, and leaf stable carbon isotope composition). We show that within both mixed and monodominant tropical forest ecosystems, the highest functional specialization and lowest functional redundancy are solely found in rare tree species and significantly more in rare species holding large‐sized individuals. Rare species cover the entire range of low and high functional redundancy, contributing both unique and redundant functions. Loss of species supporting functional redundancy could be buffered by other species in the community, including more abundant species. This is not the case for species supporting high functional specialization and low functional redundancy, which would need specific conservation attention. In terms of tropical forest management planning, we argue that specific conservation of large‐sized trees is imperative for long‐term maintenance of ecosystem functioning.

## 1 INTRODUCTION

Tropical forests host an enormous diversity of tree species (Slik et al., 2015) and are an important component of the global carbon balance (Pan et al., 2011). Even though they only cover 7%–10% of the global land area, they store ~25% of terrestrial carbon, account for ~33% of terrestrial net primary productivity (Bonan, 2008), and hold ~96% of tree species diversity (Fine, Ree, & Burnham, 2008). However, these forests face significant threats as a result of deforestation, forest degradation, and global climate change, including a continuous loss of biodiversity (Hooper et al., 2012; Naeem, Duffy, & Zavaleta, 2012).

Several recent studies propose that conservation of the forest for climate mitigation (protecting and enhancing biosphere carbon stocks) should go hand in hand with biodiversity conservation (e.g., Cavanaugh et al., 2014; Diaz, Hector, & Wardle, 2009; Poorter et al., 2015). The conservation of species should aim in the first place at preventing negative effects of biodiversity loss on ecosystem functioning, aside from the classic motivations of preserving the diversity of life or the precautionary principle (Cardinale et al., 2012; Diaz et al., 2009). Specifically, numerous studies show a positive relationship between diversity and ecosystem function, mostly studied in grasslands (e.g., Hector et al., 1999; Tilman et al., 2001; Soliveres et al., 2016), but also studies in forest ecosystems show diversity enhancing primary productivity (Zhang, Chen, & Reich, 2012 and references therein; Vilà et al., 2013; Liang et al., 2016) and standing carbon stocks (Cavanaugh et al., 2014; Poorter et al., 2017, 2015; Zhang & Chen, 2015). Two main mechanisms (not mutually exclusive) behind this relationship are postulated the following: (a) the niche complementarity effect, through which an increased resource use and nutrient retention are possible via niche differentiation or partitioning and interspecific facilitation, thus enhancing overall productivity (Loreau & Hector, 2001; Hooper et al., 2005; Tilman, 2001), and (b) the selection effect, stating that diverse communities are more likely to include one or more highly productive or high‐biomass species (Hooper et al., 2005; Loreau & Hector, 2001).

The high species diversity is maintained by a large number of rare species and few abundant species (Hubbell, 2013; ter Steege et al., 2013); community assembly theories provide insights into the coexistence of such high number of species. Niche‐based coexistence theories rely on meaningful differences in the ecological strategies of co‐occurring species (Weiher & Keddy, 1999; Wright, 2002); as opposed to neutral theory in which ecological equivalence among species is assumed (Hubbell, 2001). Within niche‐based theories, two processes are generally proposed: (1) environmental filtering, by which co‐occurring species converge in strategy as imposed by the abiotic environment (Cornwell & Ackerly, 2010; Keddy, 1992); (2) niche differentiation, by which co‐occurring species diverge in strategy mainly driven by the mechanism of resource partitioning (Silvertown, 2004). These two processes do not preclude one another (Kraft, Valencia, & Ackerly, 2008; Maire et al., 2012), with differences in traits and ecological strategies persisting through interaction with the prevailing environment (Gaston, 2011). Over time, the main driving process of community assembly could change, from initial environmental filtering due to site conditions to niche differentiation due to competition for light and other biotic interactions (Letcher et al., 2012; Lohbeck et al., 2014). Species and functional diversity should thereby increase as forest mature.

However, within established community assemblages, especially in highly diverse systems as tropical forests, little is known about the role individual species play and the relative functional importance of rare and abundant species for the biodiversity–productivity relationship (or ecosystem functioning in general) remains unclear. It has been argued that abundant species and their functional properties drive ecosystem functioning (biomass‐ratio hypothesis; Grime, 1998). Other work has shown the importance of contrasting trait values for a positive diversity effect on productivity (Zhang et al., 2012), and building on the biomass‐ratio hypothesis, diversity in the functional properties of abundant species could be needed to maintain this positive relationship. Conversely, rare species can have significant impacts on a variety of different processes (Lyons & Schwartz, 2001; Lyons, Brigham, Traut, & Schwartz, 2005 and references therein) and have the potential to support important traits (Leitão et al., 2016; Mouillot, Bellwood, et al., 2013). With rare species being specifically susceptible to loss due to natural and anthropogenic disturbances such as overexploitation, habitat degradation, or climate change (Davies, Margules, & Lawrence, 2004; ter Steege et al., 2015), quantification of their contribution to ecosystem functioning and the consequences of their loss are of particular importance.

From the perspective of biodiversity conservation as a means of maintaining ecosystem functioning and conserving and enhancing carbon storage and productivity, a better understanding of the role of individual species is necessary. In this study, we assess the functional importance of individual tree species in two highly diverse old‐growth tropical forest systems in the central Congo Basin. This functional assessment was made using functional traits related to resource acquisition, as complementarity of trait values therein could inform us on a positive influence on ecosystem productivity. Two species‐specific functional indexes were selected, functional specialization and functional redundancy, to represent which species hold the most extreme and unique combinations of traits (Leitão et al., 2016). Using this functional assessment, we investigate the following research questions: (a) What is the relationship between a species’ functional specialization or redundancy and its relative abundance? With rare species being more susceptible to loss, increased insights in the functional importance of these species are imperative. A recent study in a tropical forest in French Guiana (Leitão et al., 2016; Mouillot, Bellwood, et al., 2013) shows rare species supporting the most distinct combinations of traits, with low redundancy. Investigating if these results hold in a different tropical forest in terms of species composition, forest structure, and biogeographical conditions is particularly important to provide more insight for the need for biodiversity conservation for ecosystem functioning. (b) What is the relationship between a species’ functional specialization or redundancy and its contribution to carbon storage in the community? In terms of planning for forest carbon mitigation and biodiversity conservation, identifying if a potential relationship exists will either allow for an integrated forest management planning or highlight the need for parallel tactics in terms of management for carbon mitigation and management for biodiversity conservation. (c) Do the relationships assessed in the first two research questions vary in tropical forest communities with varying environmental filtering? That is, will similar relationships between species functional importance and abundance or contribution to carbon persist within a narrower functional space? We investigate two old‐growth forest systems with a different dominance structure in the central Congo Basin: a mixed species forest and a monodominant forest dominated by *Gilbertiodendron dewevrei* (De Wild.) J. Léonard. Monodominant *Gilbertiodendron* forest patches are naturally occurring and are found sparsely distributed across tropical Africa alongside the mixed forest (Hart, Hart, & Murphy, 1989; Peh, Sonké, Lloyd, Quesada, & Lewis, 2011), often along rivers (Fayolle et al., 2014) although not exclusively (Hart et al., 1989). The monodominance by *Gilbertiodendron *is a type of monodominance that is not clearly dependent on edaphic conditions (Peh, Sonké, et al., 2011). However, the monodominant species itself imposes strong environmental filtering by altering the abiotic environment (details can be found in Peh, Lewis, & Lloyd, 2011). The monodominant forest studied has lower species and functional diversity than the adjacent mixed forest, showing a narrower functional niche space (Kearsley et al., 2017).

## 2 METHODS

### 2.1 Study area, species selection, and trait measurement

We examined the influence of rare species on the functional diversity of tropical communities at the UNESCO Man and Biosphere reserve in Yangambi, DR Congo. The reserve covers an area of 6,297 km^2^ just north of the Congo River, and the study site is located in the southwestern part of the reserve (N00°48ʹ; E24°29ʹ). As measured in the Yangambi reserve, the region receives an annual precipitation of 1,839 ± 206 mm (1980–2012) with an average dry season length of 3.3 ± 1.3 months with monthly precipitation lower than 100 mm, during December–February. Temperatures are high and constant throughout the year with a minimum of 24.2 ± 0.4°C in July and a maximum of 25.5 ± 0.6°C in March. Soils in the Yangambi plateau are Ferralsols (WRB‐214: IUSS Working Group WRB. 2015), primarily formed from fluvio‐eolian sediments, composed mostly of quartz sand, kaolinite clay, and hydrated iron oxides.

Permanent sampling plots of one hectare were installed and measured in 2012 (Kearsley et al., 2013) in old‐growth mixed forest (*n* = 5), and old‐growth monodominant forest (*n* = 5) dominated by *Gilbertiodendron dewevrei* (De Wild.) J. Leonard. All plots within a forest type were located in a similar habitat and were situated within approximately a 5‐km and 10‐km radius from each other for mixed and monodominant forest, respectively (Supplementary Information Figure S1). Within all plots, all trees with a DBH ≥10 cm have been measured for DBH and identified to species level. Based on this inventory, a subset of species was selected for trait sampling. Within each plot, species were ranked from highest to lowest species‐specific basal area and were included for sampling until they cumulatively covered 95% of the plot‐level basal area. Next, for each selected species, the individual trees that would be sampled were selected by stratified random sampling within diameter classes of 10–20, 20–30, 30–50, and >50 cm DBH. Two individuals were randomly selected for sampling within each diameter class when possible (i.e., if present in the plot), excluding damaged trees. A total of 728 individuals were sampled, covering 90 species in the mixed forest and 82 species in the monodominant forest. Note that as such not all species in our study site have been included in trait sampling. However, we have covered a substantial amount of the rare species (i.e., species with a relative abundance <5%), accounting for, respectively, 42.9% and 68.7% in the mixed and monodominant forest communities. The list of all sampled species and number of sampled individuals can be found in Supporting Information Table S1. All samples were collected between March and May 2012.

A collection of six commonly used traits related to plant resource capture and growth were measured. The selected functional traits are wood density (WD), specific leaf area (SLA), leaf carbon content (LCC), leaf nitrogen content (LNC), leaf phosphorus content (LPC), and leaf stable carbon isotope composition (δ^13^C). SLA is part of a suite of traits associated with the leaf economics spectrum of fast‐to‐slow resource capture (Wright et al., 2004) and is correlated with primary production, carbon and nutrient cycling, and litter decomposition (Poorter, Niinemets, Poorter, Wright, & Villar, 2009). WD is often used as a key trait for biogeochemical ecosystem processes such as carbon sequestration and turnover rates (Chave et al., 2009). LNC and LPC are included in this study to reflect nutrient status. Nutrient availability has a strong effect on photosynthetic carbon gain, as both phosphorus and nitrogen availability constrain leaf photosynthetic capacity (Domingues et al., 2010). δ^13^C is measured as a proxy of the intrinsic water use efficiency, which is the ratio of photosynthetic carbon fixation to stomatal conductance (Farquhar, Ehleringer, & Hubick, 1989). Leaf and wood sampling and trait analysis were done in a standardized way following Cornelissen et al. (2003), and all methodological details can be found in Kearsley et al. (2017).

### 2.2 Species' abundance, contribution to aboveground carbon, and maximum size

Within the two forest types, we assess each species’ relative abundance, contribution to aboveground carbon (AGC), and the maximum size attained by an individual in the tree community. The relative abundance of each species is defined as the relative number of stems of that species compared to the species with the highest number of stems in the considered forest type. In the subsequent text, we will distinguish abundant, nonabundant, and rare species based on the respective thresholds of >10%, 10%–5%, and <5% of a species’ relative abundance. Statistical analysis is, however, performed in a continuous way along the rarity–commonness gradient. Note that our approach dealing with rarity–commonness is based only on local abundance, not on a restricted geographical distribution of the population. Next, each species contribution to AGC is assessed as the percentage of contribution the total AGC stock of the community. To this end, AGC for each individual tree is estimated using the allometric equation of Chave et al. (2015) for moist tropical forest including height and wood density, with biomass assumed to be 50% carbon. Site and forest type‐specific height‐diameter relationships (not species‐specific) are used to estimate height (Kearsley et al., 2013). Hereby, the weight of a species in the community is quantified as the percentage each species contributes to the total AGC stock of the community. In the subsequent text, all species contributing most to carbon storage, which together hold over 50% of the carbon stock in the forest, are classified and referred to as hyperdominant (Fauset et al., 2015; ter Steege et al., 2013), irrespective of their abundance.

Although abundance and contribution to AGC are often related, with highly abundant species often contributing significantly to total carbon storage, this is not always the case. Dependent on the size of the individuals, abundant species could contribute relatively little to total carbon storage if individual tree sizes are small, or conversely, rare species could contribute significantly to carbon by a large size. Therefore, we include the assessment of the maximum diameter (DBH; diameter at breast height) an individual of each species attained in the investigated tree communities. Note that in this case, maximum size of a species can only be interpreted as a plot‐level characteristic for this community and not as a species trait, since not all species will have attained their maximum potential size.

Species abundance, contribution to AGC, and maximum size are determined for the aggregate of all plots within each forest type, thus for the combined 5 ha within mixed and monodominant forest.

### 2.3 Functional indexes

The functional specialization of each species, that is, the mean distance of a species from the rest of the species pool in functional space (Mouillot, Graham, Villéger, Mason, & Bellwood, 2013), and the functional originality of each species, that is, the isolation of a species in the functional space occupied by a given community (Mouillot, Graham, et al., 2013), are calculated using a distance‐based approach in a multidimensional functional space. First, the Shapiro–Wilk test is used to test for normality of the individual traits and skewed distributions of LNC, LPC, and SLA are log transformed. All traits are rescaled between zero and one to ensure an equal weight of each trait within the assessment of species’ functional specialization and originality. Next, a standardized multidimensional functional space is created by scaling and centering of each trait according to all species values. The functional specialization of each species (FSpeS) is then calculated as the distance to the centroid (0,0) on the six axes of this functional space using an extension of Pythagoras’ theorem (Bellwood, Wainwright, Fulton, & Hoey, 2006). Species near to the centroid are functionally generalized, and those furthest away are most specialized (Bellwood et al., 2006). The functional originality of each species (FOriS) is calculated by estimating pair‐wise Euclidian distances between the target species and all other species in the community and subsequently determining the distance to the nearest neighbor (Mouillot, Graham, et al., 2013). A high FOriS, that is, high functional distance to its nearest neighbor, reflects how functionally isolated a species is, while a low FOriS shows the species shares their traits more closely with other species and reflects a high functional redundancy. Accordingly, since 0 ≤ FOriS ≤ 1, the complement of functional originality is used to measure the functional redundancy of a species: FRedS = 1 − ForiS (Mouillot, Graham, et al., 2013; Ricotta et al., 2016). Functional redundancy is then defined as a species' proximity to other species in the functional space occupied by a given community. All indexes were calculated using the R software (R Core Team 2016) and the function “FDchange”.

### 2.4 Statistical analysis

The relationship between species‐specific abundance, contribution to AGC, and maximum size with species‐specific functional specialization and redundancy is assessed using ordinary least square regressions. Specifically for the relationship between the relative abundance of a species and both functional indexes, preliminary analysis revealed a significant heterogeneity in the variance of the response variable (in this case, a triangular relationship). This implies that there could be more than a single slope describing the predictor–response relationship measured on a subset of these factors (Cade & Noon, 2003). Therefore, quantile regressions near the upper boundaries of the response variable FSpeS (75th and 90th quantiles) near the lower boundaries of the response variable FRedS (25th and 10th quantiles) are additionally performed allowing us to detect relevant slopes of the independent variable on the upper or lower limit of the response variables (Cade & Noon, 2003; Koenker, 2005). Quantile regressions were assessed using the “rq” function from the “quantreg” R package.

Additional preliminary analysis within the high variation found in both functional specialization and redundancy of the rare species (relative abundance <5%) revealed a gradient of species maximum size, with size increasing with functional specialization and decreasing with functional redundancy. Therefore, for different thresholds of functional specialization, we determined the mean of the maximum DBH for species with a functional specialization above this threshold (ratio of upper vs. lower boundaries provided similar results, as the mean DBH of species in the lower boundaries did not shift significantly). Similarly, for different thresholds of functional redundancy, we determined the mean of the maximum DBH for species with a functional redundancy below this threshold (ratio of upper vs. lower boundaries provided similar results, as the mean DBH of species in the upper boundaries did not shift significantly). To test whether the gradient in species size with increasing or decreasing thresholds of functional specialization and redundancy, respectively, was significant, we performed 10,000 bootstrapped randomizations with the 95% confidence interval determined using the percentile method (R package “boot”).

## 3 RESULTS

**Figure 1:**
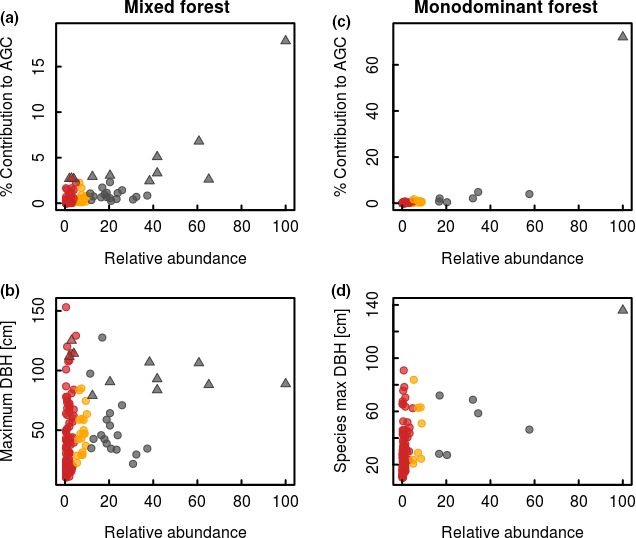
Relation between species relative abundance and contribution to aboveground carbon (AGC) (a, c) and maximum size (b, d) within the mixed forest (left column) and monodominant *Gilbertiodendron* forest (right column) at the Yangambi forest reserve, DR Congo. Each dot represents a single species, with its classification based on its abundance indicated: abundant (gray), nonabundant (yellow), and rare (red). Several species are further classified as hyperdominant in terms of their total contribution to carbon (species indicated with triangles). All species are investigated within the combined 5 ha plots of mixed and monodominant forest. The relative abundance is expressed as a percentage of the maximal observed abundance within the community. The contribution to AGC is expressed as a percentage of the total AGC stock within the community. Maximum size is expressed as the maximum DBH (diameter at breast height; cm) found within all individuals of each species

The relative abundance of a species and its percentage contribution to AGC storage of the community are positively related in both mixed forest and monodominant forest (*r* = 0.80 and 0.83, respectively, *p* < 0.001; Figure 1a,c; to test for influence of monodominant species, leave‐one‐out cross‐validation confirmed relationship), showing that abundant species are important contributors to the AGC storage. However, nonabundant/rare species include both species with small and relatively large contributions to the overall carbon stock, with three rare species even being classified as hyperdominant in terms of contributing to carbon storage in the mixed forest (Figure 1a). This is related to the size of an individual of a species, with several rare species attaining large sizes (Figure 1b,d).

**Figure 2:**
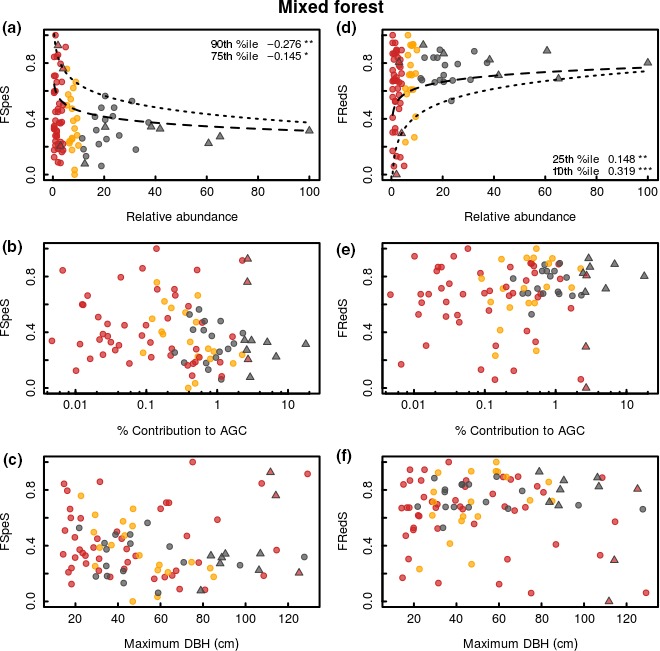
Species‐specific functional specialization (FSpeS; left column) and species‐specific functional redundancy (FRedS; right column) as a function of species relative abundance (a, d), contribution to aboveground carbon (AGC) (b, e), and maximum size (c, f) within the mixed forest at the Yangambi forest reserve, DR Congo. Each dot represents a single species, with its classification based on its abundance indicated: abundant (gray), nonabundant (yellow), and rare (red). Several species are further classified as hyperdominant in terms of their total contribution to carbon (species indicated with triangles). All species are investigated within the combined 5 ha mixed forest plots. The functional specialization of a species quantifies its uniqueness compared to all other species in the community based on a set of resource acquisition traits. The functional specialization of each species quantifies the mean distance of a species from the rest of the species pool in functional space. Functional redundancy quantifies a species’ proximity to other species in the functional space occupied by a given community. The relative abundance is expressed as a percentage of the maximal observed abundance within the community. The contribution to AGC is expressed as a percentage of the total AGC stock within the community. Maximum size is expressed as the maximum DBH (diameter at breast height; cm) found within all individuals of each species. Significant quantile regressions for FSpeS are indicated as dashed lines for the 75th quantile and as dotted lines for the 90th quantile; for FRedS dashed lines indicate significant regressions for the 25th quantile and dotted lines for the 10th quantile (ns *p* > 0.05, *p < 0.05, **p < 0.01,***p < 0.001). No significant linear regressions were found

**Figure 3:**
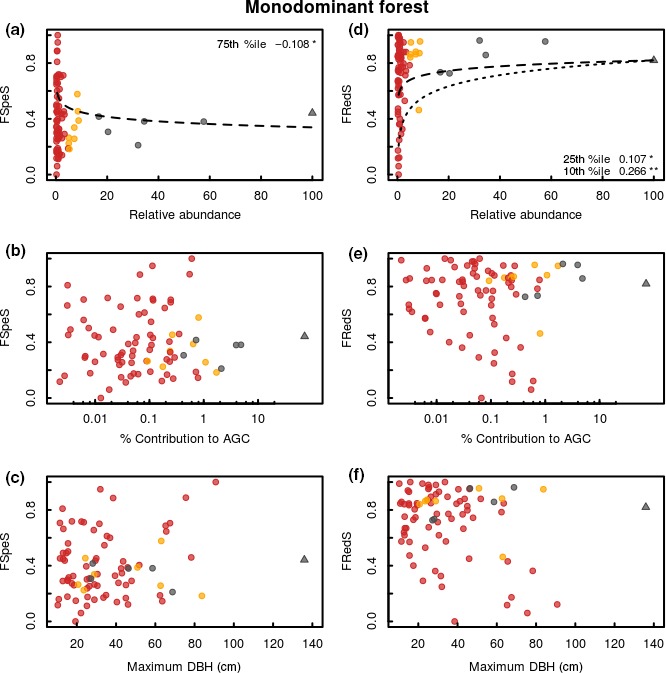
Species‐specific functional specialization (FSpeS; left column) and species‐specific functional redundancy (FRedS; right column) as a function of species relative abundance (a, d), contribution to aboveground carbon (AGC) (b, e), and maximum size (c, f) within the monodominant *Gilbertiodendron* forest at the Yangambi forest reserve, DR Congo. Each dot represents a single species, with its classification based on its abundance indicated: abundant (gray), nonabundant (yellow), and rare (red). Several species are further classified as hyperdominant in terms of their total contribution to carbon (species indicated with triangles). All species are investigated within the combined 5 ha monodominant forest plots. The functional specialization of a species quantifies its uniqueness compared to all other species in the community based on a set of resource acquisition traits. The functional specialization of each species quantifies the mean distance of a species from the rest of the species pool in functional space. Functional redundancy quantifies a species' proximity to other species in the functional space occupied by a given community. The relative abundance is expressed as a percentage of the maximal observed abundance within the community. The contribution to AGC is expressed as a percentage of the total AGC stock within the community. Maximum size is expressed as the maximum DBH (diameter at breast height; cm) found within all individuals of each species. Significant quantile regressions for FSpeS are indicated as dashed lines for the 75th quantile and as dotted lines for the 90th quantile; for FRedS dashed lines indicate significant regressions for the 25th quantile and dotted lines for the 10th quantile (ns *p* > 0.05, *p < 0.05, **p < 0.01,***p < 0.001). No significant linear regressions were found

**Figure 4:**
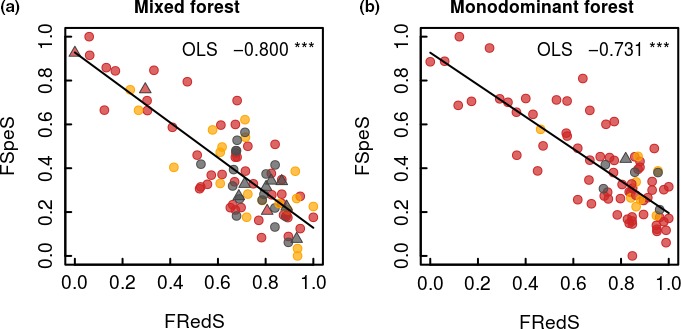
Relation between species‐specific functional specialization (FSpeS) and species‐specific functional redundancy (FRedS) for the (a) mixed forest and (b) monodominant *Gilbertiodendron* forest at the Yangambi forest reserve, DR Congo. Each dot represents a single species, with its classification based on its abundance indicated: abundant (gray), nonabundant (yellow), and rare (red). Several species are further classified as hyperdominant in terms of their total contribution to carbon (species indicated with triangles). All species are investigated within the combined 5 ha plots of mixed and monodominant forest. Ordinary least square (OLS) regressions are indicated. The functional specialization of each species quantifies the mean distance of a species from the rest of the species pool in functional space. Functional redundancy quantifies a species’ proximity to other species in the functional space occupied by a given community

The regression quantile models show that variation in functional specialization was inversely related to the relative abundance of species in both mixed forest and monodominant forest (Figures 2a and 3a; Table 1). Generally, the highest functional specialization of traits related to resource acquisition and growth is found for the rare species, while low functional specialization is found within the entire range from rare to common species. The variation in functional redundancy was positively related to the relative abundance of species in both mixed forest and monodominant forest (Figures 2d and 3d; Table 1). Generally, low functional redundancy is found for the rare species, while high functional redundancy is found within the entire range from rare to common species. Functional specialization and redundancy are negatively related in both mixed forest (−0.800; *p* < 0.001; *R* ^2^ = 0.69) and monodominant forest (−0.731; *p* < 0.001; *R* ^2^ = 0.66) (Figure 4), showing that the species holding high functional specialization in the community also show a low functional redundancy and vice versa. No significant relationships are found between the functional specialization or redundancy and contribution to AGC or the maximum size of a species in the community in either mixed and monodominant forest (Figures 2b,c,e,f and 3b,c,e,f; Table 1).

**Table 1:** Parameter values for ordinary least square (OLS) regressions for species‐specific functional specialization and functional redundancy as a function of species relative abundance, contribution to aboveground carbon (AGC), and maximum size for the mixed and monodominant *Gilbertiodendron* forest at the Yangambi forest reserve, DR Congo

		Mixed			Monodominant		
		Slope	*p*	*R* ^2^	Slope	*p*	*R* ^2^
Functional specialization v							
Relative abundance	OLS	−0.002	0.382	0.029	−0.001	0.801	0.001
	75th %ile	−0.145	0.032		−0.108	0.018	
	90th %ile	−0.276	0.002		−0.149	0.092	
Contribution to AGC	OLS	−0.01	0.386	0.008	−0.0005	0.866	0.001
Maximum size	OLS	0.0001	0.918	0.0001	0.001	0.369	0.013
Functional redundancy v							
Relative abundance	OLS	0.003	0.06	0.042	0.002	0.173	0.023
	25th %ile	0.148	0.022		0.107	0.049	
	10th %ile	0.319	0.0009		0.266	0.001	
Contribution to AGC	OLS	0.01	0.389	0.009	0.002	0.63	0.003
Maximum size	OLS	−0.0006	0.475	0.006	−0.002	0.356	0.016

Note

For functional specialization as a function of species relative abundance, parameter values are given for the 75th and 90th quantile regressions, assessed to account for the heterogeneity in the variance of the response variable functional specialization. Similarly, for functional redundancy, parameter values are given for the 10th and 25th quantile regressions.

**Figure 5:**
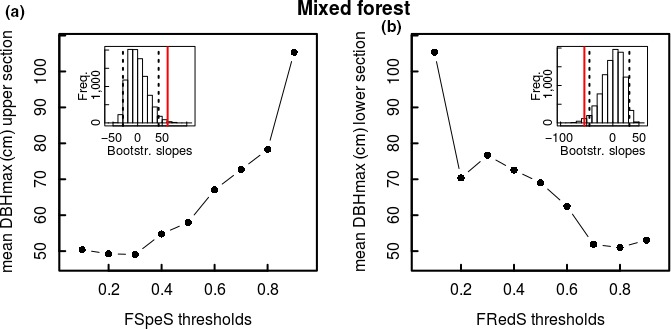
Mean maximum species size of rare species (relative abundance <5%) with (a) a species‐specific functional specialization above the different thresholds of species‐specific functional specialization (FSpeS; in steps of 0.1) and (b) a species‐specific functional redundancy below the different thresholds of species‐specific functional redundancy (FRedS; in steps of 0.1) in the mixed forest at the Yangambi forest reserve, DR Congo. In the insets, slopes of 10,000 bootstrapped randomizations, with 95th confidence interval indicated with dashed lines, and slope of current graph indicated in red.

**Figure 6:**
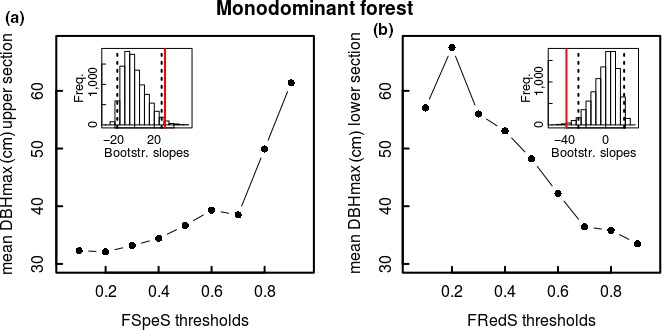
Mean maximum species size of rare species (relative abundance <5%) with (a) a species‐specific functional specialization above the different thresholds of species‐specific functional specialization (FSpeS; in steps of 0.1) and (b) a species‐specific functional redundancy below the different thresholds of species‐specific functional redundancy (FRedS; in steps of 0.1) in monodominant *Gilbertiodendron* forest at the Yangambi forest reserve, DR Congo. In the insets, slopes of 10,000 bootstrapped randomizations, with 95th confidence interval indicated with dashed lines, and slope of current graph indicated in red.

A detailed analysis within the group of the rare species shows that species maximum size increases significantly with increased functional specialization and decreased functional redundancy in both mixed forest and monodominant forest (Figures 5 and 6). Looking into detail, in the mixed forest, only 15 species show a high functional specialization (arbitrary threshold of >0.6), 6 of which are rare species with large‐sized individuals with a maximum DBH of ≥70 cm (threshold set by Slik et al., 2013 for classification as “large trees”), 2 of which are even classified as hyperdominant in terms of their contributing to carbon storage. This is almost half (46.2%) of rare species holding large‐sized individuals (13 in total) that show a high functional specialization. These six species also all hold a low functional redundancy of <0.3 (on the scale of 0 to 1). In the monodominant forest, only 17 species show a high functional specialization (>0.6) of which two species have a maximum DBH ≥70 cm. These two species also both show very low functional redundancies of 0.06 and 0.12, respectively, on the scale of 0 to 1. Considering that in the entire tree community, besides the monodominant *Gilbertiodendron dewevrei* itself, only four species hold individuals reaching a DBH of ≥70 cm, this shows that also in this forest community, a high ratio of the species holding large‐sized individuals has a high functional specialization and low functional redundancy.

## 4 DISCUSSION

Understanding the role of individual species for the relationship between biodiversity and ecosystem productivity and/or standing biomass can aid in formulating more detailed conservation plans, specifically in highly diverse ecosystems. At our study area, we show that high functional specialization and low functional redundancy in traits related to resource acquisition are only found in several rare species, and specifically in a disproportionately high ratio of rare species holding large‐sized individuals. Functional redundancy was found in all abundant species and the majority of nonabundant/rare species.

### 4.1 Rare species holding large‐sized individuals support high functional specialization and low redundancy

High functional specialization and low redundancy are only found in rare species at our tropical forest study site. This shows that these rare species are found at the extremities of the functional space, where they show uniqueness in their set of trait values (Mouillot, Graham, et al., 2013). By supporting these distinct and complementary set of traits, these rare species are more likely to also support complementary functions in the community in terms of resource utilization (Hooper et al., 2005; Leitão et al., 2016; Mouillot, Villéger, Scherer‐Lorenzen, & Mason, 2011; Mouillot, Bellwood, et al., 2013). These species may thereby increase resource use efficiency in this community, and by this way of niche complementarity enhance productivity in this forest community (van Ruijven & Berendse, 2005).

Moreover, we show a higher proportion of rare species with large‐sized individuals holding functional specialization and originality as compared to rare species holding smaller‐sized individuals. These distinct rare species with large‐sized individuals could potentially play a larger role in ecosystem functioning as those with smaller‐sized individuals as proposed by the mass‐ratio hypothesis (Grime, 1998). Together with the abundant species, they represent the majority of standing biomass, with a high impact on ecosystem properties such as productivity, carbon sequestration, water relations, and nutrient cycling (Grime, 1998; Slik et al., 2013).

### 4.2 Functional redundancy in majority of rare species

Based on their high occurrence, abundant species support functions highly influencing main ecosystem functioning. The majority of rare species in our study show high redundancy in their functional trait combinations related to resource acquisition, potentially indicating they contribute little to ecosystem functioning or stability. In the context of biodiversity enhancing productivity and standing biomass (and biodiversity conservation with this aim), saturation of productivity gain with increased diversity may occur due to functional redundancy (Hooper et al., 2005; Naeem, Bunker, Hector, Loreau, & Perrings, 2009). Moreover, we cannot state that all functionally distinct rare species contribute to this relationship, as it is unclear where saturation with increased diversity would occur. Studies on the diversity–productivity/biomass relationships in forest ecosystems have mainly been performed in relatively low‐diverse systems such as boreal and temperate forests or plantations (Gamfeldt et al., 2013; Vilà et al., 2013; Zhang et al., 2012), showing a saturation with few species (six to eight species). On the other hand, Liang et al. (2016) revealed a consistent positive biodiversity–productivity relationship across forests worldwide (although relatively few tropical sites), with no saturation effect found despite a concave‐down pattern (reporting average tree species richness of 5.79 (*SD* 8.64) per plot (size 0.04 ha (*SD* 0.12)). In highly diverse tropical forests, no direct study is available (to our knowledge) addressing this saturation effect. However, investigating this relationship at different spatial scales in tropical forests, recent studies found that the positive effect of diversity on standing biomass was strongest at small spatial scales (Chisholm et al., 2013; Poorter et al., 2015; Sullivan et al., 2017). At these smaller scales (0.04 ha, 0.1 ha), relatively few species could profit from an additional species in terms of niche complementarity, while at larger scales (1 ha), this effect could saturate.

In our study, the functional specialization and redundancy of a species are determined compared to all other species in the tree community at a larger scale, not just to neighboring trees. At smaller scales, other species could be identified as functionally specialized or distinctive and with local niche complementarity. However, the finding that rare species with large‐sized individuals hold proportionately high functional specialization and low redundancy at the community level could be important at this larger spatial scale. Their high contribution in standing biomass (and productivity) combined with high complementarity in resource use could be crucial for an effect of increased diversity at these scales. Functionally, distinct rare species with smaller‐sized individuals potentially do not contribute much at these larger scales, but would be highly significant at smaller scales.

### 4.3 Niche differentiation in rare species in both mixed and monodominant forests

Although our study is not set up to study community assembly in these highly diverse tropical forests, our finding of linking species abundance with functional redundancy could relate to aspects of niche‐based coexistence theory. The high functional specialization and low functional redundancy supported by rare species indicate that niche differentiation may be an important mechanism for sustaining the large diversity in this tropical forest. Resource partitioning may be an important mechanism here, for rare species diverging from other species in the community, specifically from more common species, in a set of trait values related to resource acquisition. On the other hand, high functional redundancy and low specialization found specifically in abundant but also in many rare species, indicates convergence in trait values potentially related to environmental filtering. Simultaneous occurrence of converging and diverging strategies could drive community assembly in diverse systems (Kraft et al., 2008; Maire et al., 2012), and shift in importance over time (Letcher et al., 2012; Lohbeck et al., 2014). Moreover, even in the monodominant *Gilbertiodendron* forest where a stronger environmental filter invokes a narrower functional space (Kearsley et al., 2017; Peh, Lewis, et al., 2011), niche differentiation through resource partitioning remains an important mechanism for coexistence of rare species and sustaining a high diversity.

### 4.4 Limitations of the study

Some limitations of our study have to be kept in mind. Firstly, the study area sampled is relatively small, presenting 5 ha for each of the two forest types. The species rarity–commonness is therefore only shown locally, and more spatially distributed data are needed to present rarity or commonness at a larger geographical scale. However, since our results corroborate findings from studies from different geographical tropical regions (Leitão et al., 2016; Mouillot, Bellwood, et al., 2013) with highly varying species composition (Slik et al., 2015), we believe our main conclusion is sound. Secondly, we assume that the traits we present represent a species’ function in the community. It, however, needs to be acknowledged that functional traits are merely proxies for ecological functions, and functions are not measured directly. Thirdly, and related to the previous limitation, we only investigated the functional importance of species based on traits related to resource acquisition. Detailed investigation into multifunctionality of species would be critical, since it could identify other ecological functions of species.

### 4.5 Importance of rare species conservation

Biodiversity loss is an important threat in tropical forests with a high number of tree species risking population loss or extinction (ter Steege et al., 2015), although data on net species loss for tropical forest remain limited (Gonzalez et al., 2016). Investigating the functional importance of individual species in a tropical forest, we show a high proportion of functional similarity and redundancy in species, which might act as a buffer against species loss (Gaston & Fuller, 2008). This functional redundancy might insure ecosystem functioning through replacement with other (more abundant) species following biodiversity erosion (Fonseca & Ganade, 2001; Yachi & Loreau, 1999). However, we also found that species showing high functional specialization and low redundancy are exclusively rare, corroborating the findings of Mouillot, Bellwood, et al. (2013) and Leitão et al. (2016) in a tropical forest site in French Guiana. If our assumption that functional specialization and distinctiveness translates to distinct and complementary functions is valid, these species play an important role in ecosystem functioning, irrespective of their low abundance. Loss of these rare species could therefore have important implications for the maintenance of ecosystem functioning. Unfortunately, Mouillot, Bellwood, et al. (2013) found that these rare species and the functions they hold are likely to be the most vulnerable. Conservation of rare species should therefore be an important aspect for the maintenance of ecosystem functioning. Moreover, rare species holding large‐sized individuals are often species with commercial importance (at our site, e.g., *Pericopsis elata* (Harms) Meeuwen (Afrormosia or African Teak)). Caution is therefore especially important during forest management practices, which could highly benefit from functional characterization research.

Our findings show that rare species holding large‐sized individuals contribute disproportionately to ecosystem functioning, both in terms of standing biomass and complementary traits in terms of resource acquisition. Large old trees support a wide range of important ecological functions (as reviewed in Lindenmayer & Laurance, 2017), including hydrological regimes, nutrient cycles, and numerous ecosystem processes, and contain a significant proportion of the stand carbon within a few individuals (Bastin et al., 2015; Slik et al., 2013). Large old trees are, however, vulnerable to numerous threats, resulting in an observed global decline (Lindenmayer, Laurence, & Franklin, 2012). We therefore emphasize that specific attention should be paid to species holding large‐sized individuals in conservation planning.

## 5 CONCLUSION

We conclude that high functional specialization and low redundancy are supported by a fraction of the rare species in the tree community, more specifically in a high proportion of rare species holding large‐sized individuals. Within the context of forest conservation for carbon mitigation initiatives, conservation of these rare species for long‐term maintenance of ecosystem functioning is crucial. Prioritizing conservation effort to functionally distinct rare species is, however, challenging due to large efforts that would be needed for their characterization and identification, and overall biodiversity conservation should be the aim. However, even in cases where limited capacity for biodiversity conservation would be available, our findings show that conservation of rare species holding large‐sized individuals could be an important starting point.

In conclusion, we show that biodiversity conservation in tropical forest ecosystems is necessary beyond the classic motivations of preserving the diversity of life or the precautionary principle. Similar results to our African study are reported within the Neotropical forest (Mouillot, Bellwood, et al., 2013) invoking a pantropical conservation policy, irrespective of the different dynamics in both tropical forests and highlighting the cobenefit of safeguarding functional diversity within carbon mitigation projects.

## CONFLICT OF INTEREST

None declared.

## AUTHOR CONTRIBUTIONS

HV, PB, and DH developed the overall project. EK, KH, HV, PB, and DH designed this research study. EK and KH performed the research. EK collected data. EK and KH analyzed the data. EK wrote the manuscript. All authors contributed to the interpretation of results and implications and provided comments on the manuscript.

## Data Availability

All trait data are available in the TRY Plant Trait Database (www.try-db.org). Vegetation data are available in ForestPlots.Net (www.forestplots.net) and through sPlot—The Global Vegetation Database.
