# Supplementary material for: Large‐sized rare tree species contribute disproportionately to functional diversity in resource acquisition in African tropical forest
Source: Ecol Evol. 2019 Apr 2;9(8):4349–61. doi: 10.1002/ece3.4836 (PMC6476792; doi:10.1002/ece3.4836)
Supplement: Supplementary file 1 [file ECE3-9-4349-s001.docx]

**Supplementary information**


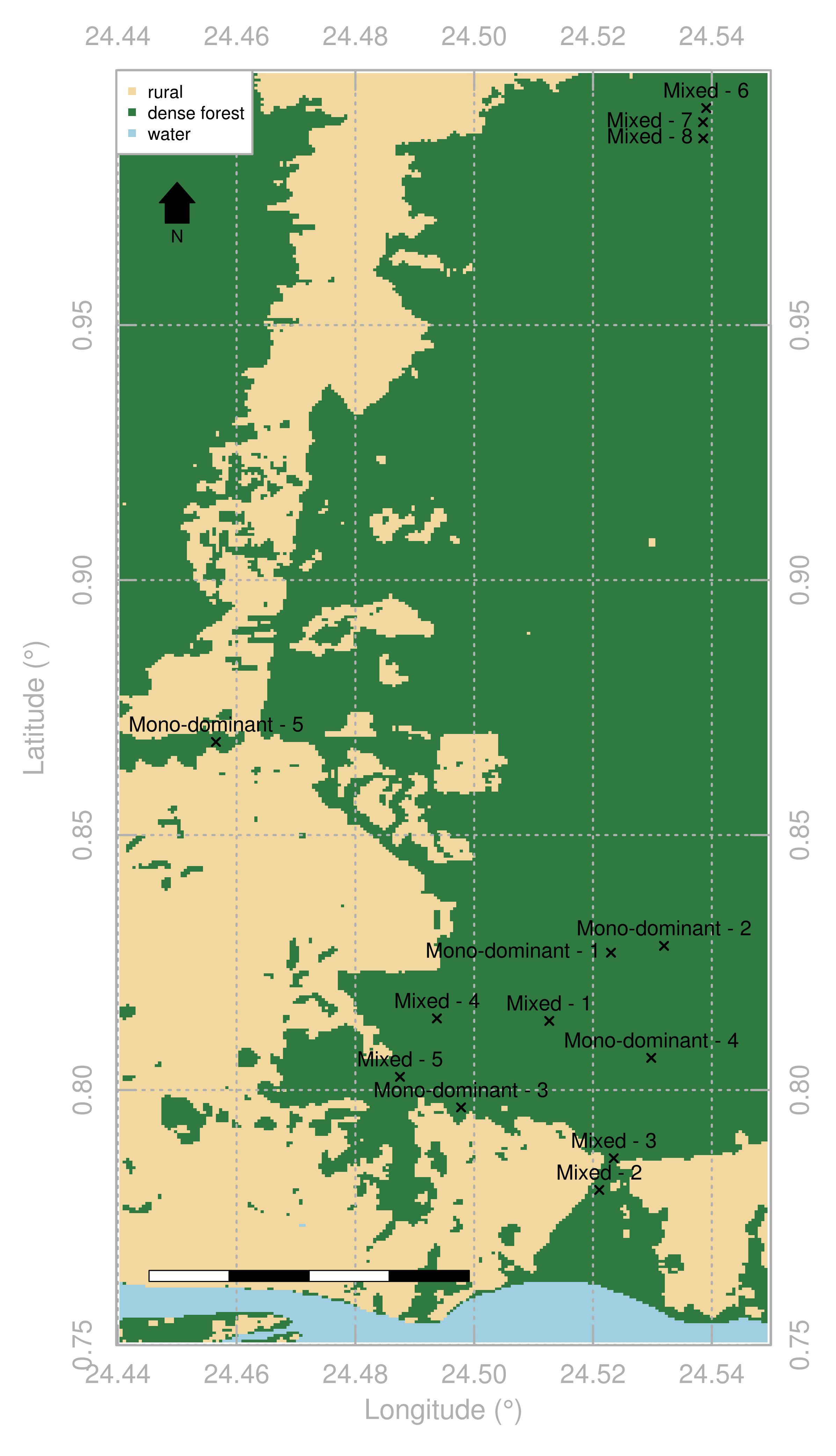
**Figure S1:** Plot distribution of mixed and mono-dominant forest plots in the Yangambi forest reserve, DR Congo. Green areas denote forested areas with a forest cover >= 50%, light brown areas denote areas with a forest cover < 50% and blue areas denote water bodies. Map data is based upon moderate resolution vegetation continuous field calibrated Landsat data with a spatial resolution of ~60m as described by Hansen et al. (2008) (http://globalmonitoring.sdstate.edu/projects/congo/). In the scale bar, one block equals 1.5 km.

Hansen, M. C. et al. (2008). A method for integrating MODIS and Landsat data for systematic monitoring of forest cover and change in the Congo Basin. *Remote Sens. Environ*. **112**, 2495–2513.

**Table S1:** List of species sampled within the mixed and monodominant old-growth forest. All trait data are available in the TRY Plant Trait Database (www.try-db.org).

| **Family** | **Species** | **Individuals sampled** |
| --- | --- | --- |
| Apocynaceae | Alstonia boonei De Wild. | 1 |
| Fabaceae | Angylocalyx pynaertii De Wild. | 8 |
| Annonaceae | Anonidium mannii (Oliv.) Engl. & Diels | 19 |
| Anacardiaceae | Antrocaryon nannanii De Wild. | 2 |
| Fabaceae | Aphanocalyx cynometroides Oliv. | 3 |
| Fabaceae | Baikiaea insignis Benth. | 6 |
| Zygophyllaceae | Balanites wilsoniana Dawe & Sprague | 1 |
| Sapindaceae | Blighia welwitschii (Hiern) Radlk. | 4 |
| Burseraceae | Canarium schweinfurthii Engl. | 1 |
| Meliaceae | Carapa procera DC. | 20 |
| Euphorbiaceae | Cavacoa quintasii (Pax & K.Hoffm.) J.Léonard | 16 |
| Cannabaceae | Celtis mildbraedii Engl. | 10 |
| Cannabaceae | Celtis tessmannii Rendle | 11 |
| Sapotaceae | Chrysophyllum africanum A. DC. | 9 |
| Sapotaceae | Chrysophyllum lacourtianum De Wild. | 11 |
| Sapindaceae | Chytranthus sp. | 1 |
| Phyllanthaceae | Cleistanthus caudatus Pax | 17 |
| Myristicaceae | Coelocaryon preussii Warb. | 15 |
| Malvaceae | Cola griseiflora De Wild. | 12 |
| Malvaceae | Cola sp. | 3 |
| Rubiaceae | Colletoecema dewevrei (De Wild.) E.M.A.Petit | 7 |
| Combretaceae | Combretum lokele Liben | 11 |
| Rubiaceae | Craterispermum cerinanthum Hiern | 2 |
| Fabaceae | Cynometra hankei Harms | 5 |
| Burseraceae | Dacryodes edulis (G.Don) H.J.Lam | 1 |
| Burseraceae | Dacryodes osika (Guillaumin) H.J.Lam | 2 |
| Fabaceae | Dialium excelsum Steyaert | 2 |
| Fabaceae | Dialium sp. | 2 |
| Fabaceae | Dialium pachyphyllum Harms | 27 |
| Ebenaceae | Diospyros chrysocarpa F. White | 3 |
| Ebenaceae | Diospyros crassiflora Hiern | 9 |
| Ebenaceae | Diospyros iturensis (Gürke) Letouzey & F.White | 6 |
| Putranjivaceae | Drypetes gossweileri S. Moore | 12 |
| Putranjivaceae | Drypetes sp. Group A | 4 |
| Putranjivaceae | Drypetes sp. Group B | 9 |
| Putranjivaceae | Drypetes sp. Group C | 2 |
| Meliaceae | Entandrophragma candollei Harms | 2 |
| Meliaceae | Entandrophragma sp. | 1 |
| Meliaceae | Entandrophragma utile (Dawe & Sprague) Sprague | 1 |
| Fabaceae | Erythrophleum suaveolens (Guill. & Perr.) Brenan | 6 |
| Bignoniaceae | Fernandoa adolfi-friderici Gilg & Mildbr. | 3 |
| Clusiaceae | Garcinia chromocarpa Engl. | 2 |

**Table S1** continued

| **Family** | **Species** | **Individuals sampled** |
| --- | --- | --- |
| Clusiaceae | Garcinia punctata Oliv. | 25 |
| Clusiaceae | Garcinia sp. | 1 |
| Fabaceae | Gilbertiodendron dewevrei (De Wild.) J.Leonard | 33 |
| Fabaceae | Gilletiodendron mildbraedii (Harms) Vermoesen | 4 |
| Annonaceae | Greenwayodendron suaveolens (Engl. & Diels) Verdc. | 15 |
| Salicaceae | Homalium sp. | 2 |
| Irvingiaceae | Irvingia excelsa Mildbr. | 6 |
| Irvingiaceae | Irvingia grandifolia (Engl.) Engl. | 5 |
| Annonaceae | Isolona thonneri (De Wild & Th. Dur.) Engl. & Diels | 11 |
| Meliaceae | Leplaea cedrata (A. Chev.) E.J.M. Koenen & J.J. de Wilde | 3 |
| Meliaceae | Leplaea thompsonii (Sprague & Hutch.) E.J.M.Koenen & J.J.de Wilde | 12 |
| Chrysobalanaceae | Maranthes glabra (Oliv.) Prance | 3 |
| Pandaceae | Microdesmis yafungana J.Léonard | 11 |
| Fabaceae | Millettia drastica Welw. Ex Baker | 2 |
| Annonaceae | Monodora angolensis Welw. | 2 |
| Urticaceae | Musanga cecropioides R.Br. ex Tedlie | 6 |
| Salicaceae | Oncoba flagelliflora (Mildbr.) S. Hul | 1 |
| Olacaceae | Ongokea gore (Hua) Pierre | 8 |
| Sapindaceae | Pancovia harmsiana Gilg | 10 |
| Sapindaceae | Pancovia laurentii (De Wild.) Gilg ex De Wild. | 10 |
| Pandaceae | Panda oleosa Pierre | 14 |
| Fabaceae | Paramacrolobium coeruleum (Taub.) J. Leonard | 3 |
| Fabaceae | Pentaclethra macrophylla Benth. | 5 |
| Lecythidaceae | Petersianthus macrocarpus (P.Beauv.) Liben | 11 |
| Fabaceae | Piptadeniastrum africanum (Hook.f.) Brenan | 2 |
| Fabaceae | Prioria balsamifera (Vermoesen) Breteler | 6 |
| Fabaceae | Prioria buchholzii (Harms) Breteler | 8 |
| Fabaceae | Prioria oxyphylla (Harms) Breteler | 2 |
| Fabaceae | Pterocarpus soyauxii Taub. | 5 |
| Myristicaceae | Pycnanthus angolensis (Welw.) Warb. | 10 |
| Simaroubaceae | Quassia undulata (Guill. & Perr.) D.Dietr. | 12 |
| Euphorbiaceae | Ricinodendron heudelotii (Baill.) Heckel | 1 |
| Violaceae | Rinorea angustifolia (Thouars) Baill. subsp. engleriana (De Wild. & T.Durand) Grey-Wilson | 4 |
| Violaceae | Rinorea oblongifolia (C.H.Wright) C.Marquand ex Chipp | 6 |
| Fabaceae | Scorodophloeus zenkeri Harms | 35 |
| Achariaceae | Scottellia coriacea A. Chev. ex Hutch. & Dalziel | 3 |
| Myristicaceae | Staudtia kamerunensis Warb. var. gabonensis (Warb.) Fouilloy | 13 |
| Malvaceae | Sterculia sp. | 1 |
| Malvaceae | Sterculia dawei Sprague | 1 |
| Olacaceae | Strombosia grandifolia Hook.f. ex Benth. | 12 |
| Olacaceae | Strombosia pustulata Oliv. | 4 |
| Olacaceae | Strombosiopsis tetrandra Engl. | 9 |
| Sapotaceae | Synsepalum subcordatum De Wild. | 10 |
| Myrtaceae | Syzygium guineense (Willd.) DC. | 1 |

**Table S1** continued

| **Family** | **Species** | **Individuals sampled** |
| --- | --- | --- |
| Fabaceae | Tessmannia africana Harms | 15 |
| Fabaceae | Tessmannia sp. | 1 |
| Fabaceae | Tetrapleura tetraptera (Schum. & Thonn.) Taub. | 1 |
| Meliaceae | Trichilia gilgiana Harms | 6 |
| Meliaceae | Trichilia gilletii De Wild. | 1 |
| Meliaceae | Trichilia heudelotii Planch. ex Oliv. | 14 |
| Meliaceae | Trichilia prieuriana A.Juss. | 6 |
| Meliaceae | Trichilia sp. | 1 |
| Sapotaceae | Tridesmostemon omphalocarpoides Engl. | 19 |
| Moraceae | Trilepisium madagascariense DC. | 6 |
| Meliaceae | Turraeanthus africana (Welw. ex C.DC.) Pellegr. | 10 |
| Rutaceae | Vepris louisii G.C.C.Gilbert | 1 |
| Lamiaceae | Vitex ferruginea Schumach. & Thonn. | 10 |
